# Retrospective study of sonographic findings in bone involvement associated with rotator cuff calcific tendinopathy: preliminary results of a case series*

**DOI:** 10.1590/0100-3984.2014.0077

**Published:** 2015

**Authors:** Marcello H. Nogueira-Barbosa, Everaldo Gregio-Junior, Mario Muller Lorenzato

**Affiliations:** 1 PhD, Associate Professor of Radiology at Ribeirão Preto Medical School, University of São Paulo (FMRP-USP), Ribeirão Preto, SP, Brazil.; 2 MD, Radiologist, Fellow at Hospital das Clínicas da Faculdade de Medicina de Ribeirão Preto da Universidade de São Paulo (HCFMRP-USP), Ribeirão Preto, SP, Brazil.; 3 MD, Radiologist at Radiologia Especializada Ribeirão Preto, Ribeirão Preto, SP, Brazil.

**Keywords:** Ultrasonography, Computed tomography, Calcific tendonitis, Rotator cuff, Shoulder

## Abstract

**Objective:**

The present study was aimed at investigating bone involvement secondary to rotator cuff calcific tendonitis at ultrasonography.

**Materials and Methods:**

Retrospective study of a case series. The authors reviewed shoulder ultrasonography reports of 141 patients diagnosed with rotator cuff calcific tendonitis, collected from the computer-based data records of their institution over a four-year period. Imaging findings were retrospectively and consensually analyzed by two experienced musculoskeletal radiologists looking for bone involvement associated with calcific tendonitis. Only the cases confirmed by computed tomography were considered for descriptive analysis.

**Results:**

Sonographic findings of calcific tendinopathy with bone involvement were observed in 7/141 (~ 5%) patients (mean age, 50.9 years; age range, 42-58 years; 42% female). Cortical bone erosion adjacent to tendon calcification was the most common finding, observed in 7/7 cases. Signs of intraosseous migration were found in 3/7 cases, and subcortical cysts in 2/7 cases. The findings were confirmed by computed tomography. Calcifications associated with bone abnormalities showed no acoustic shadowing at ultrasonography, favoring the hypothesis of resorption phase of the disease.

**Conclusion:**

Preliminary results of the present study suggest that ultrasonography can identify bone abnormalities secondary to rotator cuff calcific tendinopathy, particularly the presence of cortical bone erosion.

## Abstract (pt)

**Objetivo:**

Investigar o envolvimento ósseo secundário a tendinopatia calcificada do manguito rotador por meio da ultrassonografia.

**Materiais e Métodos:**

Estudo retrospectivo de uma série de casos. Foi realizada busca dos relatórios de ultrassonografia do ombro no sistema informatizado da instituição, no período de quatro anos. Cento e quarenta e um pacientes foram diagnosticados com tendinopatia calcificada do manguito rotador. Os achados de imagem foram analisados retrospectivamente em consenso por dois radiologistas musculoesqueléticos experientes. Os casos sem confirmação pela tomografia computadorizada foram excluídos da análise descritiva.

**Resultados:**

Foram identificados sinais de tendinopatia calcificada com envolvimento ósseo pela ultrassonografia em 7/141 (~ 5%) dos pacientes (idade: 50,9 anos; faixa etária: 42-58 anos; 42% do sexo feminino). Erosão do osso cortical adjacente à calcificação tendínea foi o achado mais comum, observado em 7/7 casos. Foram identificados sinais de migração intraóssea em 3/7 casos e cistos subcorticais adjacentes à calcificação tendínea em 2/7 casos. Os achados foram confirmados pela tomografia computadorizada. Nenhuma calcificação associada com anormalidades ósseas mostrou sombra acústica, favorecendo a fase reabsortiva da doença.

**Conclusão:**

Nossos resultados preliminares sugerem que a ultrassonografia é capaz de identificar anormalidades ósseas secundárias a tendinopatia calcificada do manguito rotador, especialmente a presença de erosões da cortical óssea.

## INTRODUCTION

Calcific tendinopathy is related to deposition of calcium hydroxyapatite in tendons. However, the origin of this micro crystal deposition disease remains obscure. Rotator cuff calcification occurs in approximately 2.5 to 7.5% of the shoulders in healthy patients^(1)^. Tendon calcifications are more commonly diagnosed by means of radiography, however, ultrasonography is also a reliable technique in the detection and localization of rotator cuff calcifications^(2,3)^. Ultrasonography is also useful to identify whether the tendon calcification is hard or soft^(2,3)^.

Calcific tendinopathy is a self-limited condition, and spontaneous resorption of calcified deposits might occur^(4)^. Calcific tendinopathy eventually cause focal resorption of adjacent cortical bone and intraosseous migration of calcic material might occur^(5-9)^. There is little available information in the literature on the possible usefulness of ultrasonography in the detection of bone changes associated with calcific tendinopathy^(10,11)^.

The present study aims to describe ultrasonography findings in bone involvement secondary to rotator cuff calcific tendinopathy.

## MATERIALS AND METHODS

Retrospective study of a series of cases approved by the Institutional Review Board (IRB). Informed written consent was waived by the IRB due to the retrospective nature of the study. A search on the Radiology Information System databank of the institution was carried out over a period of four years, seeking for the terms "*tendinopatia*" (tendinopathy), "*tendinite*" (tendonitis), "*calcificada*" (calcific), "*hidroxiapatita*" (hydroxyapatite) and "*cristais*" (crystals) in ultrasonography reports in order to identify cases of calcific tendinopathy.

Ultrasonography was part of routine investigation of shoulder pain on patients at the authors' university hospital. The ultrasonography scans were performed with a HD 11 apparatus (Philips Medical Systems; Bothell, WA, USA), utilizing a 3-12 MHz transducer, or with a Logiq-E apparatus (GE Medical System; Jiangsu, China) utilizing a 5-13 MHz transducer. In the ultrasonography documentation protocol, images from the subscapular, supraspinatus and infraspinatus tendons are routinely acquired in the longitudinal and transverse axes of such tendons, respectively. The long head tendon of the biceps tendon is routinely imaged in the longitudinal and transverse axes in the regions of the rotator interval and of the intertubercular groove. In cases where any focal abnormality of any tendon is identified, it is documented in at least two orthogonal planes. Sonographic sections of the acromioclavicular joint, glenohumeral joint, spinoglenoid notch and suprascapular fossa and infrascapular fossa are also documented routinely.

The imaging findings of calcific tendinopathy were consensually reviewed by two musculoskeletal radiologists. Each radiologist had over ten-year experience in musculoskeletal ultrasonography at the moment of the review. Bone erosion was defined as a rupture in the surface of the cortical bone identified in images from at least two orthogonal planes. Subcortical cysts were identified as hypoechoic well defined focal abnormality with rounded contours in subcortical bone.

The cases were included in the analysis of secondary bone involvement only when computed tomography (CT) images were available to serve as a reference. The mean time interval between ultrasonography and CT scans in this study was approximately 2.6 days, ranging from 1 to 10 days.

## RESULTS

The search for reports at the Hospital Information System was retrospective and identified 2,547 patients submitted to shoulder ultrasonography. The search on the ultrasonography reports detected 141/2,547 cases (approximately 5.5%) diagnosed with rotator cuff calcific tendinopathy. All evaluated patients with findings of bone compromising at ultrasonography were symptomatic.

Signs of calcific tendinopathy with involvement of adjacent bone were identified by ultrasonography in 7/141 cases (approximately 5% of the patients), with four being men and 3 being women, with ages ranging between 42-58 years (mean age of 50.9 years). CT confirmed the bone abnormalities in all of such cases. Three cases that presented sonographic findings of intraosseous involvement secondary to calcific tendinopathy were excluded, because CT image was not available for comparison.

**Figure 1:**
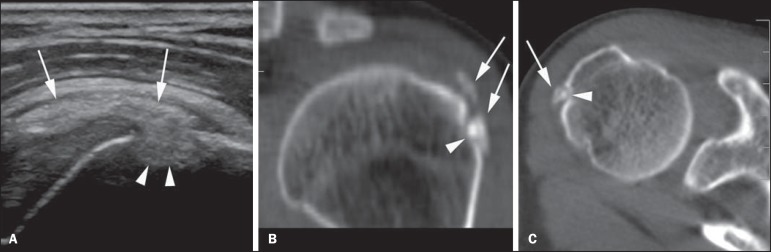
Male, 56-year-old patient. **A:** Ultrasonography in the long axis of the infraspinatus tendon showing intratendinous calcification (arrows) associated with focal bone lesion and intraosseous calcification migration (arrowheads). **B,C:** Axial CT section and oblique coronal reconstruction, respectively, confirm the presence of calcification of the infraspinatus tendon in the region of transition to the supraspinatus tendon (arrows) and adjacent cortical bone erosion (arrowheads).

**Figure 2:**
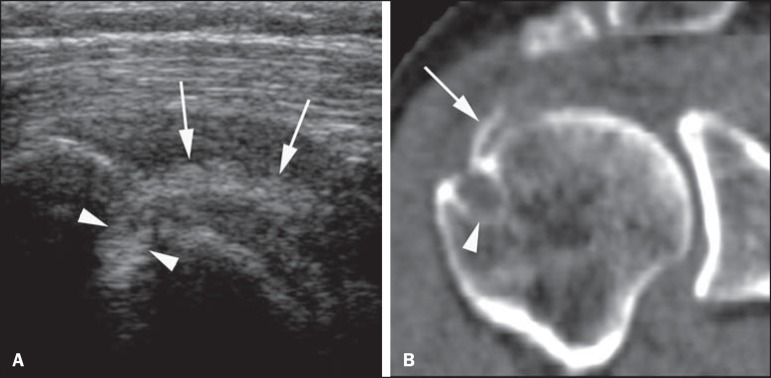
Male, 56-year-old patient. **A**: Sonographic image in the long axis of rotator cuff tendons presenting with calcification (arrows) in the supraspinatus/infraspinatus transition. Intraosseous migration is indicated by the arrowheads **B:** Coronal CT reconstruction confirms the findings. The arrow indicates calcification, and the arrowhead indicates the cystic subcortical bone lesion in the rotator cuff insertion.

**Figure 3:**
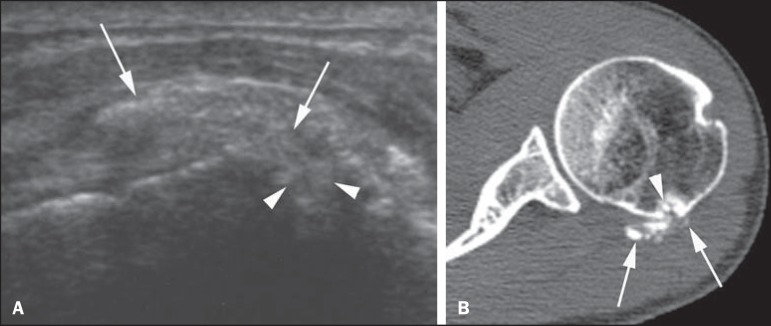
Female, 50-year-old patient. **A:** Sonographic image of the shoulder in the long axis of the infraspinatus tendon, identifying hyperechoic focal amorphous calcifications (arrows) with intraosseous migration (arrowheads) in the infraspinatus tendon. **B:** Axial CT image showing that calcifications (arrows) are at the infraspinatus tendon insertion, extending towards the greater tubercle of humerus. Bone erosion and intraosseous migration (arrowheads) are also confirmed by CT.

Tendons with calcification associated with bone abnormalities were the supraspinatus (4/7 cases) and the infraspinatus (3/7 cases). The most common sonographic finding in the series was the presence of an elongated hyperechoic intratendinous focus with adjacent bone erosion (Figures 1, 2 and 3). Acoustic shadowing was not identified in any of the calcific tendinopathy cases with bone involvement. Computed tomography confirmed erosion of the cortical bone adjacent to calcifications of the rotator cuff in all cases. Subcortical bone cysts were identified at CT in two cases, and intraosseous calcification migration was confirmed by CT in three of the cases.

## DISCUSSION

Intraosseous migration of calcium crystal deposits associated with calcific tendinopathy is well known in the literature^(5-9)^. The present study results suggest that ultrasonography can demonstrate bone involvement secondary to calcific tendinopathy, and the most common findings in this situation were demonstrated by utilizing this imaging method. In the present study, the typical presentation of calcific tendinopathy with bone involvement at ultrasonography was the presence of hyperechogenic focus without acoustic shadowing on the tendon insertion adjacent to a cortical bone erosion.

In the present study, no cases of tendinous calcifications associated with bone erosion presented acoustic shadowing. The absence of acoustic attenuation may be considered as an indication that the calcific deposits present a liquid or pasty consistency^(3)^. Therefore, our results reinforce the hypothesis that the calcifications migration from the tendon to the bone occurs in the resorption phase of the disease.

Involvement of bone marrow, cortical erosion and soft tissue edema may be secondary to calcific tendinopathy and mimic more aggressive diseases such as neoplasia or infection^(6)^. Magnetic resonance imaging and CT are considered the best methods to demonstrate involvement of bone marrow in calcific tendinopathy^(6)^. Bone involvement and intraosseous calcification migration may occur in hydroxyapatite deposition disease even in other anatomic regions, for example in intervertebral discs^(12)^.

In the present investigation, the presence of cortical bone erosion adjacent to calcification was the most important and consistent sonographic finding and was confirmed in all cases included by further investigation with CT. Intraosseous calcifications migration was unequivocally demonstrated at CT images in 3/7 cases. In the present study, ultrasonography was followed by supplementary investigation with CT within no more than 10 days. Calcific deposits associated with calcific tendinopathy may be rapidly and spontaneously resorbed^(13)^.

Generally, subcortical cystic changes may be identified in the rotator cuff insertion area, especially in the anterior region of the supraspinatus and subscapularis tendons^(14,15)^. On the other hand, cystic changes may also be associated with intraosseous migration in rotator cuff calcific tendinopathy, and focal calcifications may be observed within the cyst^(9,16)^. Subcortical cysts were identified by ultrasonography in two of the cases of the present study, demonstrating close spatial relationship between calcifications and with bone erosion. Subcortical cysts were confirmed in those two cases by CT images.

Ultrasonography is an effective imaging method in the evaluation of rotator cuff abnormalities, as well as of other shoulder abnormalities^(17)^, but there is little available data on its utilization in the detection of bone involvement secondary to calcific tendinopathy^(10,11)^.

The literature emphasizes the utilization of ultrasonography in rheumatology for the investigation of arthritis^(18-20)^. More recently, ultrasonography was utilized for the study of micro crystals deposit diseases. Ultrasonography is useful to identify deposition of crystals in gout arthritis ^(21-23)^ and in the study of tendon involvement by gout^(24,25)^. The evaluation of enthesopathy in patients presenting with fibromyalgia can also be performed by ultrasonography^(26)^. Our results are important to highlight the necessity of including hydroxyapatite deposition disease in the differential diagnosis of other enthesopathies.

The main limitation of the present study was its retrospective nature. There was a bias in the selection of patients submitted to CT. Only cases with a suspicion of bone involvement at ultrasonography were evaluated since CT is not routinely performed for further investigation of rotator cuff calcific tendinopathy. Therefore, we could estimate the diagnostic performance of ultrasonography in the detection of bone involvement in calcific tendinopathy. In addition, due to the restricted number of patients in this case series, we performed only a descriptive analysis. A prospective study with a higher number of cases should be undertaken for a better evaluation of sensitivity, specificity and effectiveness of ultrasonography in the identification of bone involvement secondary to calcific tendinopathy.

The morphology of calcific deposits at ultrasonography images presents good correlation with the clinical symptoms of calcific tendinopathy^(27)^. In the study developed by Chiou et al., calcifications without acoustic shadowing - those in the resting and resorptive phases -, were those which correlated better with clinical symptoms^(27)^.

Our preliminary results suggest that calcification migration and bone involvement can be consistently identified by ultrasonography, and reinforce the concept that bone involvement occurs in the resorption phase of calcific tendinopathy. The radiologist must identify the findings of bone involvement secondary to calcific tendinopathy at ultrasonography, due to its potential of association with pain, a feature reinforced by the present case series, and must also recognize that eventual bone erosion may occur in the enthesis as a part of the disease.
